# Nogo-B promotes angiogenesis and improves cardiac repair after myocardial infarction via activating Notch1 signaling

**DOI:** 10.1038/s41419-022-04754-4

**Published:** 2022-04-05

**Authors:** Yanjun Zheng, Jingrong Lin, Dingsheng Liu, Guoqing Wan, Xuefeng Gu, Jian Ma

**Affiliations:** 1 Shanghai Key Laboratory of Molecular Imaging, Zhoupu Hospital, Shanghai University of Medicine and Health Sciences, Shanghai, 201318 China; 2 Department of Hypertension, Ruijin Hospital, Shanghai Institute of Hypertension, Shanghai Jiao Tong University School of Medicine, Shanghai, China; 3 Department of Cardiology, Shanghai Jiao Tong University Affiliated Sixth People’s Hospital, No. 600. Yi Shan Road, Shanghai, 200233 China

**Keywords:** Heart failure, Ischaemia

## Abstract

Nogo-B (Reticulon 4B) is reportedly a regulator of angiogenesis during the development and progression of cancer. However, whether Nogo-B regulates angiogenesis and post-myocardial infarction (MI) cardiac repair remains elusive. In the present study, we aimed to explore the role and underlying mechanisms of Nogo-B in cardiac repair during MI. We observed an increased expression level of Nogo-B in the heart of mouse MI models, as well as in isolated cardiac microvascular endothelial cells (CMECs). Moreover, Nogo-B was significantly upregulated in CMECs exposed to oxygen-glucose deprivation (OGD). Nogo-B overexpression in the endothelium via cardiotropic adeno-associated virus serotype 9 (AAV9) with the mouse endothelial-specific promoter *Tie2* improved heart function, reduced scar size, and increased angiogenesis. RNA-seq data indicated that Notch signaling is a deregulated pathway in isolated CMECs along the border zone of the infarct with Nogo-B overexpression. Mechanistically, Nogo-B activated Notch1 signaling and upregulated Hes1 in the MI hearts. Inhibition of Notch signaling using a specific siRNA and γ-secretase inhibitor abolished the promotive effects of Nogo-B overexpression on network formation and migration of isolated cardiac microvascular endothelial cells (CMECs). Furthermore, endothelial Notch1 heterozygous deletion inhibited Nogo-B-induced cardioprotection and angiogenesis in the MI model. Collectively, this study demonstrates that Nogo-B is a positive regulator of angiogenesis by activating the Notch signaling pathway, suggesting that Nogo-B is a novel molecular target for ischemic disease.

## Introduction

Acute myocardial infarction (AMI) caused by coronary ischemia is the leading cause of death worldwide. After an MI, dead cardiomyocytes are replaced by fibrous scar tissues, inducing ventricular remodeling and heart failure (HF) [1, 2]. To save the ischemic myocardium, the timely rescue of cardiac blood flow in MI patients is the current therapeutic option. Angiogenesis is essential for tissue repair or regeneration, including MI [3–5]. Therapeutic angiogenesis is a potential strategy to foster new vessel formation in the ischemic peri-infarct border zone and subsequently improve post-infarction ventricular remodeling and cardiac function. To date, therapeutic approaches other than endovascular treatment are barely available to restore blood flow in ischemic tissues. In the treatment of ischemic heart disease, therapeutic angiogenesis has been extensively explored. The latter plays a fundamental role in promoting myocardial infarction repair and preventing adverse ventricular remodeling [6–8]. Therefore, identifying key regulators of angiogenesis as therapeutic targets to promote blood flow recovery after MI is of great interest.

Reticulin (RTN) is a class of protein family mainly located in the endoplasmic reticulin (ER) of cells through its ER targeting motif. Nogo-B is one of the isotypes in the RTN4 family and was previously reported to be widely expressed in vascular cells and cardiomyocytes in vivo and has various cell types in vitro [9, 10]. Recently, studies exposed that Nogo-A was undetectable while Nogo-B was expressed in the cardiac vasculature under physiological conditions. Nogo-B has been shown to control vascular function by inhibiting endothelial sphingolipid homeostasis [11]. The expression of Nogo-B was upregulated and further promoted the proliferation of hepatocytes and liver regeneration [12]. Nogo-B expressed in Kupffer cells promoted M1/M2 polarization, thereby facilitating alcoholic liver disease [13]. However, whether Nogo-B can also stimulate or modulate angiogenesis after MI remains unknown.

Notch signaling is conserved and involved in cellular growth, survival, and differentiation. The interaction between Notch receptors and their ligands leads to proteolytic cleavage and releases the Notch intracellular domain (NICD). The NICD then translocates to the nucleus to activate the transcription of target genes. Indeed, Notch signaling is closely associated with cardiovascular diseases [14, 15]. Especially during cardiac ischemia, activated Notch signaling is involved in promoting angiogenesis and alleviating I/R injury [16–19]. Additionally, activated Notch1 signaling also mediates the cardioprotection afforded by ischemic preconditioning and postconditioning [20, 21]. However, whether Nogo-B regulates Notch signaling in MI models remains to be investigated.

Herein, we analyzed the effect of Nogo-B on MI and explored the role of Notch signaling in mediating the cardioprotective effect of Nogo-B in MI in vitro and in vivo. With loss-of-function and gain-of-function strategies, results indicated that Nogo-B was sharply increased after myocardial infarction and that the cardiotropic expression of Nogo-B through a cardiotropic adeno-associated viral vector improved cardiac function, reduced scar size, and increased angiogenesis. Conversely, in vitro knockdown and inhibition of the Notch1 pathway and in vivo endothelial Notch1 heterozygous deletion reversed Nogo-B-afforded cardioprotection. These findings demonstrate that Nogo-B may also have therapeutic implications for promoting regeneration in the context of ischemic vascular disease.

## Results

### Nogo-B was increased in CMECs isolated from MI mouse hearts

**Fig. 1:**
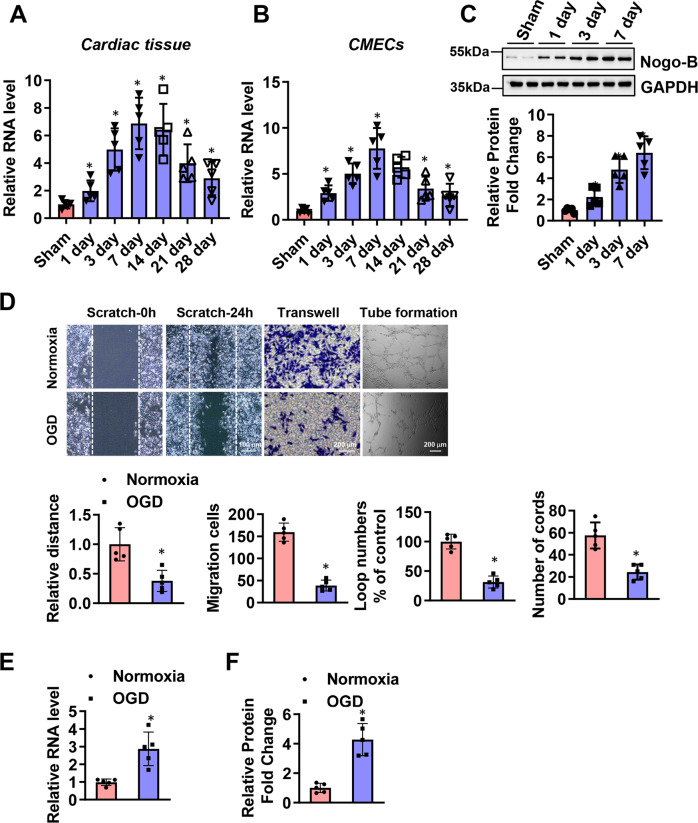
Nogo-B was increased in CMECs after MI. **A**, **B** Time course of the relative mRNA level of Nogo-B measured by Q-PCR in cardiac tissues and CMECs in the infarct border zone after MI (*n* = 5). **C** Representative and quantitative Nogo-B protein level in CMECs in the infarct border zone after MI (*n* = 5). **D** Representative and quantitative analysis of scratch, transwell, and tube formation images under normoxic or OGD conditions (*n* = 5). **E**, **F** mRNA and protein levels of Nogo-B after normoxic or OGD treatment (*n* = 5). Data were expressed as mean ± SEM. ^∗^*P* < 0.05 versus sham or normoxic group. Unpaired Student’s *t*-test (**D**–**F**), one-way ANOVA (**A**–**C**).

To evaluate the function of Nogo-B, the expression level of Nogo-B in cardiac tissues and the microcirculation after MI was examined. MI provoked an increase in Nogo-B expression, which peaked on day 7 (Fig. 1A). More evidence was acquired by isolating CMECs from sham and MI mouse hearts. Immunofluorescence was used to identify the cultured CMECs, while flow cytometry was utilized to identify the purity of cultured CMECs ((Supplementary Fig. 1). Similarly, compared with the sham group, higher expression levels of Nogo-B were found in CMECs isolated from the border zone, with the level peaking on day 7 (Fig. 1B, C). To provide further evidence of the role of Nogo-B in regulating CMECs functions and repair, in vitro experiments were performed on CMECs exposed to OGD. Moreover, the migratory capacity of CMECs was evaluated by scratch and migration assays. Exposure to OGD significantly reduced the healing area and the number of migratory CMECs (Fig. 1D). Furthermore, the in vitro tube formation ability of CMECs was significantly impaired with OGD (Fig. 1C). Meanwhile, exposure to OGD significantly increased Nogo-B expression (Fig. 1E, F). These data suggest that the endogenous increase of Nogo-B in CMECs may have clinical significance and may be involved in the pathophysiology of MI.

### Nogo-B improved cardiac function and reduced scar size

**Fig. 2:**
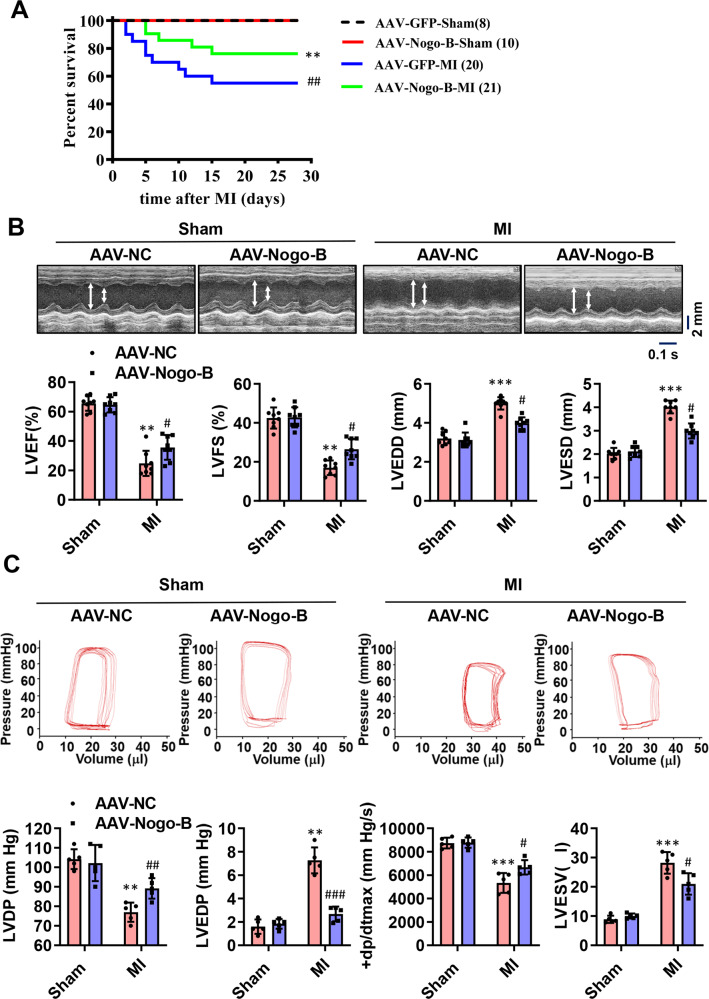
Nogo-B treatment improved cardiac functions. **A** The survival rate of sham and MI mice injected with AAV during the 4-week follow-up period. The log-rank test demonstrated significant differences between the two survival curves. **B** LVEF, LVFS, LVEDD, and LVEDS were measured by echocardiography (*n* = 8). **C** LVDP, LVEDP, LV maximum ascending rates of pressure (+dp/dt max), and LVESV measured by the pressure–volume loop at day 28 post-MI (*n* = 5). Data were expressed as mean ± SEM. ^*^P < 0.05, ^**^P < 0.01, ^***^P < 0.001 versus AAV-NC group. ^#^*P* < 0.05, ^##^*P* < 0.01, ^###^*P* < 0.001 versus AAV-NC-MI group. two-way ANOVA (**B**, **C**).

**Fig. 3:**
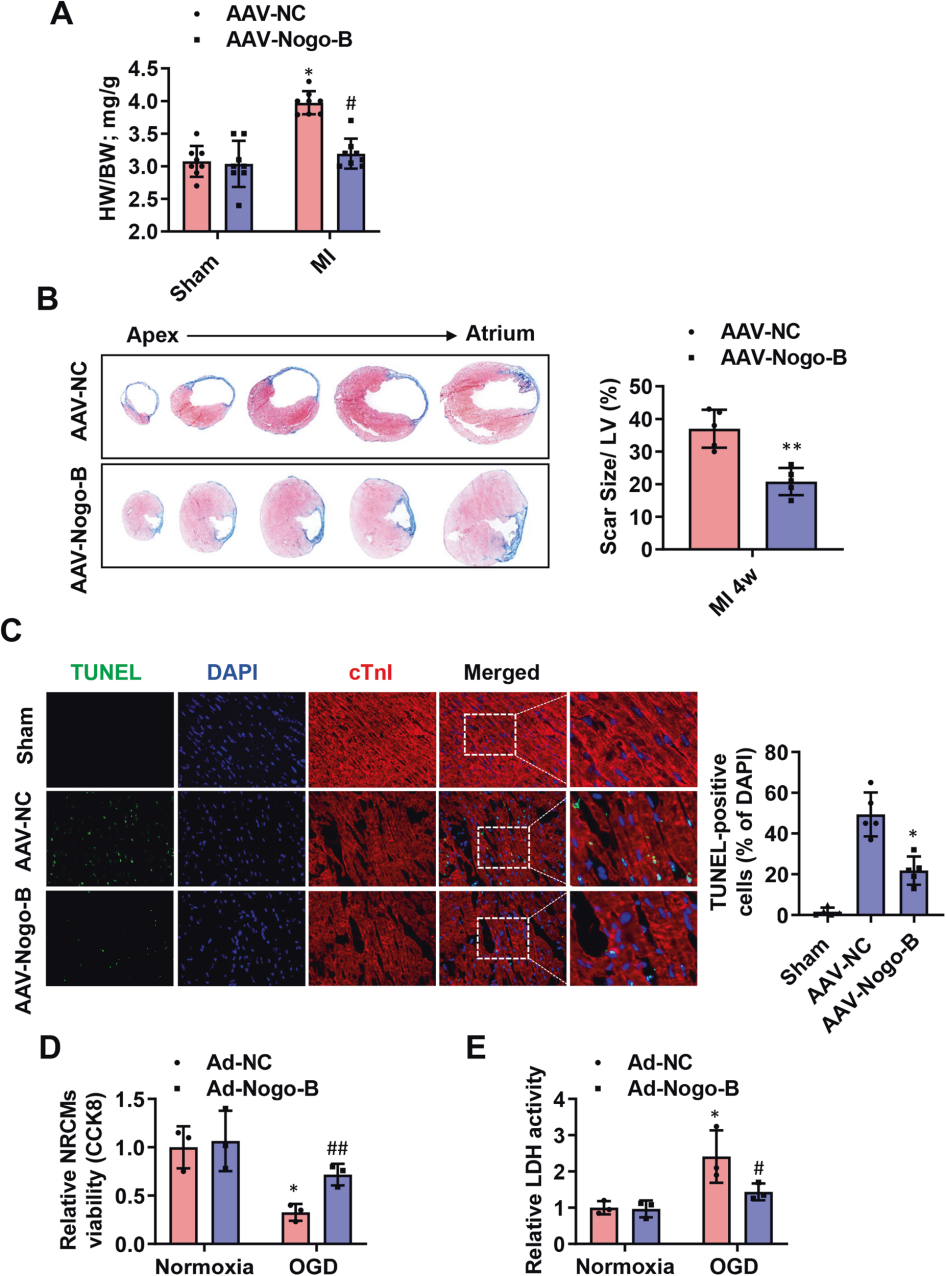
Nogo-B decreased fibrosis and increased angiogenesis after MI. **A** The heart weight to body weight ratio (*n* = 8). **B** Representative cross-sectional images and quantitative data of hearts stained with Masson’s trichrome at day 28 post-MI (*n* = 5). **C** Representation and quantification of IHC staining for TUNEL + cardiomyocytes in the border zone of infarcted hearts at day 3 post-MI (*n* = 5). ^*^P < 0.05, ^**^P < 0.01, ^***^P < 0.001 versus AAV-NC group. ^#^*P* < 0.05, ^##^*P* < 0.01, ^###^*P* < 0.001 versus AAV-NC-MI group. **D** The viability of NRCMs was measured by the Cell Counting Kit-8 (CCK8) in the medium (*n* = 3). **E** Fold changes of LDH activities in the NRCMs medium. ^*^P < 0.05 versus Ad-NC group (*n* = 3). ^#^*P* < 0.05, ^##^*P* < 0.01 versus Ad^-^NC-OGD group. Data were expressed as mean ± SEM^.^ Unpaired Student’s *t*-test (**B**), two-way ANOVA (**A** and **C**–**E**).

To determine whether Nogo-B improves cardiac function after MI, we generated a recombinant AAV cassette with Nogo-B expression driven by a mouse Tie2 promoter (AAV-Nogo-B), ensuring that the endothelial-specific expression and functional outcome were compared on day 28 post-MI. A dose-dependent effect of AAV administration was observed in CMECs, and the concentration of 1 × 10^12^ viral particles resulted in a >13-fold increase in Nogo-B expression and was thus selected for subsequent experiments. Moreover, AAV-Nogo-B injection results in significantly increased expression of Nogo-B in endothelial cells, while has little effects on the expression of Nogo-B in cardiomyocytes and cardiac fibroblasts (Supplementary Fig. 2). The death rate of MI mice did not significantly differ between the sham groups during the 4-week follow-up period, but it was significantly reduced in the AAV-Nogo-B-MI group compared to the AAV-NC-MI group (Fig. 2A). Cardiac functional parameters such as left ventricular ejection fraction (LVEF) and LV fractional shortening (LVFS) were both decreased in the AAV-NC-MI group; however, they were significantly improved in the AAV-Nogo-B-MI group (Fig. 2B). Hemodynamic alterations were further analyzed in a terminal study at day 28 post-MI using a pressure–volume loop. The MI-induced deterioration in LV developed pressure (LVDP), LV end-diastolic pressure (LVEDP), and LV maximum ascending rates of pressure (+dp/dt max) were significantly improved in the AAV-Nogo-B-MI group (Fig. 2C). Besides, the heart weight to body weight ratio was also decreased in the AAV-Nogo-B-MI group compared to the AAV-NC-MI group (Fig. 3A). Consistently, all MI animals exhibited scar formation at day 28 post-MI, as analyzed by Masson’s trichrome staining, while the scar size in AAV-Nogo-B hearts was smaller than that in the AAV-NC-MI group (Fig. 3B). Next, we analyzed the apoptosis of cardiomyocytes in the border zone of hearts on day 1 post-MI. TUNEL^+^ cardiomyocytes (Fig. 3C) were significantly attenuated in the Nogo-B overexpression group compared with the control group. These data further reinforce the hypothesis that Nogo-B overexpression not only improves LV function but also prevents the progressive deterioration of LV remodeling. To investigate whether the cardioprotective effects of Nogo-B are related to cardiomyocytes, we simulated OGD injury in the neonatal rat cardiomyocytes (NRCMs). Notably, OGD injury decreased the viability of NRCMs (Fig. 3D), and increased LDH release (Fig. 3E) was ameliorated by Nogo-B overexpression.

### Nogo-B promoted angiogenesis in vivo and in vitro

**Fig. 4:**
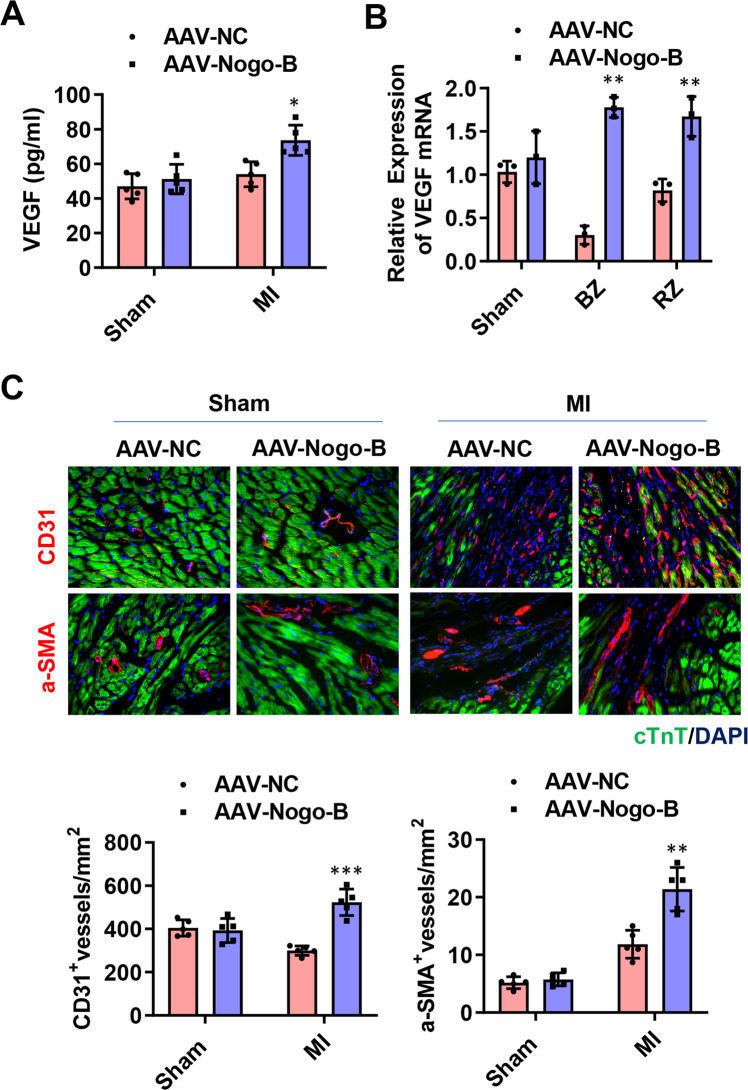
Nogo-B promoted angiogenesis after MI. **A** The mRNA level of VEGF at day 28 post-MI (*n* = 5). **B** The protein level of VEGF at day 28 post-MI in border (BZ) and remote zone (RZ) of infarcted hearts. **C** Immunochemistry staining for CD31 + endothelial cells and α-SMA + vessels at day 28 post-MI (*n* = 5). Data were expressed as mean ± SEM. ^*^P < 0.05, ^**^P < 0.01, ^***^P < 0.001 versus AAV-NC group. two-way ANOVA (**A**–**C**).

Considering that angiogenesis also contributes to the decrease in cell death and fibrotic size in ischemic hearts [22, 23], the secretion of the angiogenic factor VEGF, the number of CD31^+^ endothelial cells, and α-SMA^+^ vessels in the MI hearts were investigated. On day 28 post-MI, the upregulation of VEGF by Nogo-B overexpression was confirmed at both mRNA and protein levels (Fig. 4A, B). Besides, immunohistological chemistry (IHC) displayed increased angiogenesis (Fig. 4C, left panel) and vascular vessel formation (Fig. 4C, right panel) in the infarct and border zones of AAV-Nogo-B-MI hearts compared with the NC.

**Fig. 5:**
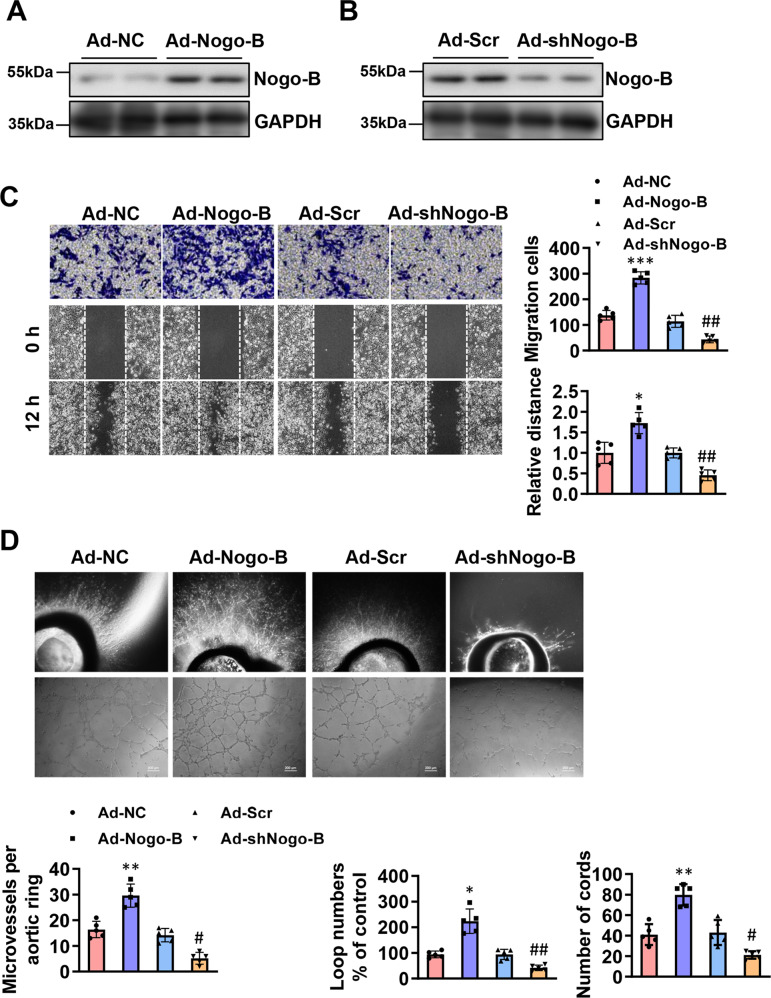
Nogo-B promoted angiogenesis in vitro. **A** The expression efficiency of Ad-Nogo-B measured by Western blot (*n* = 4). **B** The downregulation efficiency of Ad-siNogo-B measured by Western blot (*n* = 4). **C** Transwell and scratch/wound assays and tube formation analysis demonstrated that Nogo-B promoted cellular migration of ECs (*n* = 5). **D** Effect of Ad-Nogo-B and Ad-shNogo-B on vessel outgrowth in aortic rings and ECs tube formation (*n* = 5). Data were expressed as mean ± SEM. ^*^P < 0.05, ^**^P < 0.01, ^***^P < 0.001 versus Ad-NC group. ^#^*P* < 0.05, ^##^*P* < 0.01 versus Ad-Scr group. one-way ANOVA (**C**, **D**).

To further evaluate the role of Nogo-B on endothelial cells, adenoviruses expressing Nogo-B cDNA (Ad-Nogo-B) and short hairpin RNA (Ad-shNogo-B) were constructed. The change in the expression level of Nogo-B in CMECs was analyzed by western blot (Fig. 5A, B). CMECs migration was promoted in the Ad-Nogo-B group, whereas Ad-shNogo-B inhibited this effect compared to the Ad-Scramble group (Fig. 5C). Aortic rings were infected with Ad-NC, Ad-Nogo-B, Ad-scramble, or Ad-shNogo-B. The overexpression of Nogo-B increased aortic outgrowth of capillary sprouts, loop numbers, and cord number of CMECs, while Ad-shNogo-B inhibited these effects compared to the Ad-Scramble group (Fig. 5D). These data signify that Nogo-B may promote angiogenesis in AAV-Nogo-B-treated infarcted hearts.

### Nogo-B overexpression upregulated the Notch signaling pathway

**Fig. 6:**
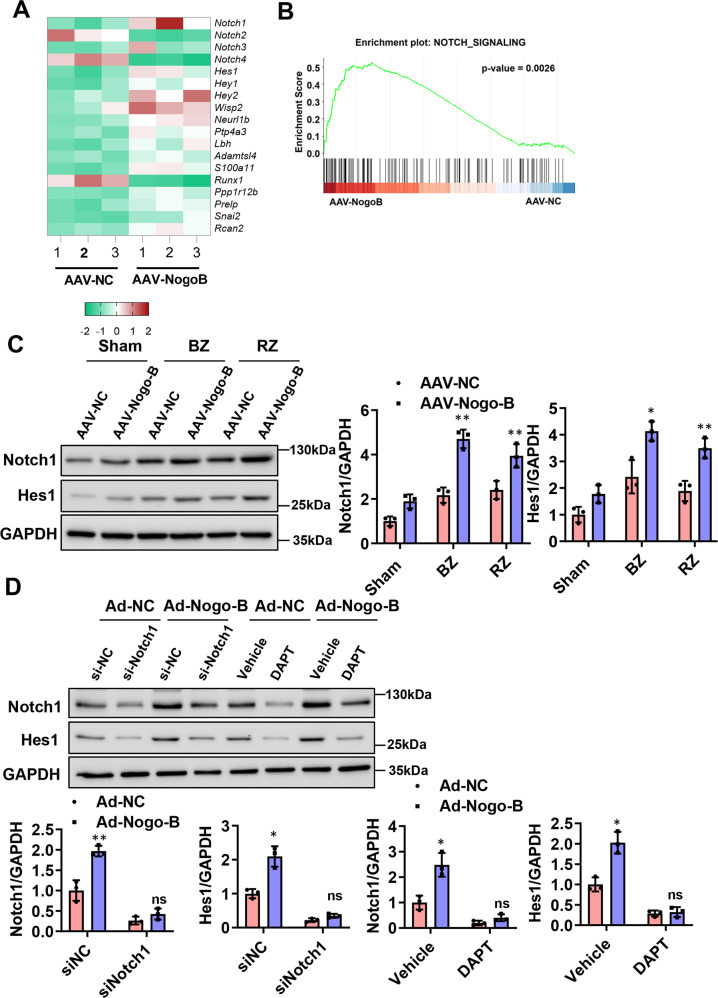
Nogo-B overexpression upregulated the Notch signaling pathway. **A** Heat map of genes related to the Notch pathway with RNA-seq for CMECs from AAV-NC or AAV-Nogo-B-infected mice after MI. **B** GSEA analyses of the gene set upregulated by Nogo-B overexpression in CMECs. **C** Protein levels of Notch and Hes1 in the AAV-Nogo-B and AAV-NC infected hearts after MI in border (BZ) and remote zone (RZ) of infarcted hearts (*n* = 5). ^*^P < 0.05, ^**^P < 0.01 versus sham group. **D** Protein levels of Notch and Hes1 in ECs treated with DAPT and siNotch (*n* = 5). ^*^P < 0.05, ^**^P < 0.01 versus Ad-NC group. ns^,^ not significant. Data were expressed as mean ± SEM. Two-way ANOVA (**C**, **D**).

To seek downstream effectors by which Nogo-B regulates angiogenesis, the RNA-seq data of CMECs isolated from AAV-Nogo-B-treated infarcted hearts versus AAV-NC-treated infarcted hearts were analyzed 7 days after the MI. It was observed that Notch-associated genes were regulated by Nogo-B (Fig. 6A, B). Western blot results showed that the protein levels of Notch1 and Hes1 gradually increased within the first week after MI compared to the sham group (Sup Fig. 3). Similarly, the expression levels of Notch1 and Hes1 were also increased in the hearts of the AAV-Nogo-B-MI group compared to the AAV-NC-MI group (Fig. 6C). To examine the function of Notch signaling in this process, CMECs were treated with a γ-secretase inhibitor, *N*-[*N*-(3,5-difluorophenyl-l-alanyl)]-d-phenylglycine *t*-butyl ester (DAPT), to block Notch cleavage and activation. Interestingly, both DAPT treatment and Notch1 knockdown by the siRNA blocked Nogo-B-induced upregulation of Notch1 and Hes1 in CMECs (Fig. 6D). Taken together, the above data demonstrated that Nogo-B activated the Notch signaling pathway in endothelial cells and hearts after MI.

### Inhibition of Notch pathway abolished Nogo-B-improved EC network formation and migration

**Fig. 7:**
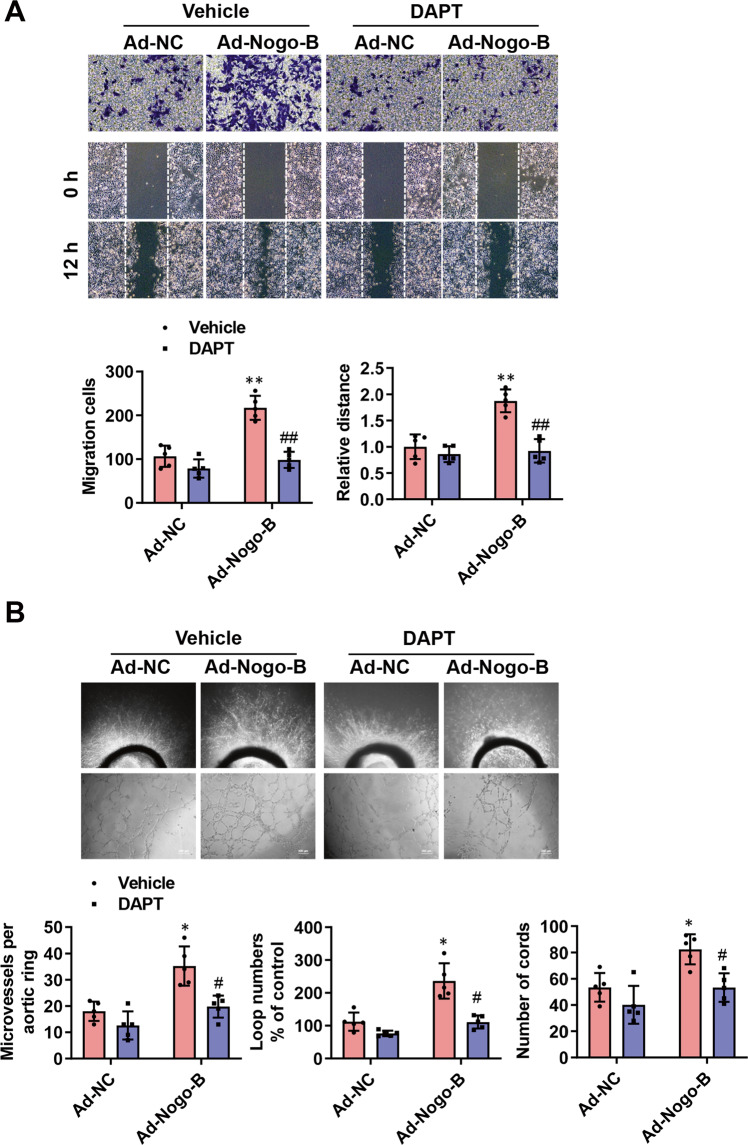
Notch pathway inhibition abolished Nogo-B overexpression in ECs network formation and migration. **A** DAPT treatment on migration, invasion, and tube formation in Ad-NC and Ad-Nogo-B-infected ECs (*n* = 5). **B** DAPT treatment on the effect of Ad-Nogo-B and Ad-NC on vessel outgrowth in aortic rings and ECs tube formation (*n* = 5). Data were expressed as mean ± SEM. ^*^P < 0.05, ^**^P < 0.01 versus Ad-NC group. ^#^*P* < 0.05, ^##^*P* < 0.01 versus Ad-Nogo-B group^.^ two-way ANOVA (**A**, **B**).

Afterward, the effects of DAPT treatment, as well as Notch1 knockdown with a specific siRNA on migration, invasion, and tube formation of CMECs, were investigated using Ad-NC and Ad-Nogo-B infected CMECs. DAPT treatment and Notch1 knockdown suppressed the promotive migratory and invasive phenotypes of Nogo-B overexpression in CMECs (Fig. 7A and Supplementary Fig. 3a). Consistently, DAPT treatment and Notch1 knockdown also reduced cellular network/capillary formation of Nogo-B-overexpressed aortic rings and ECs (Fig. 7B and Supplementary Fig. 3b).

### Notch pathway inhibition abolished the cardioprotective effects of Nogo-B in MI-injured hearts

**Fig. 8:**
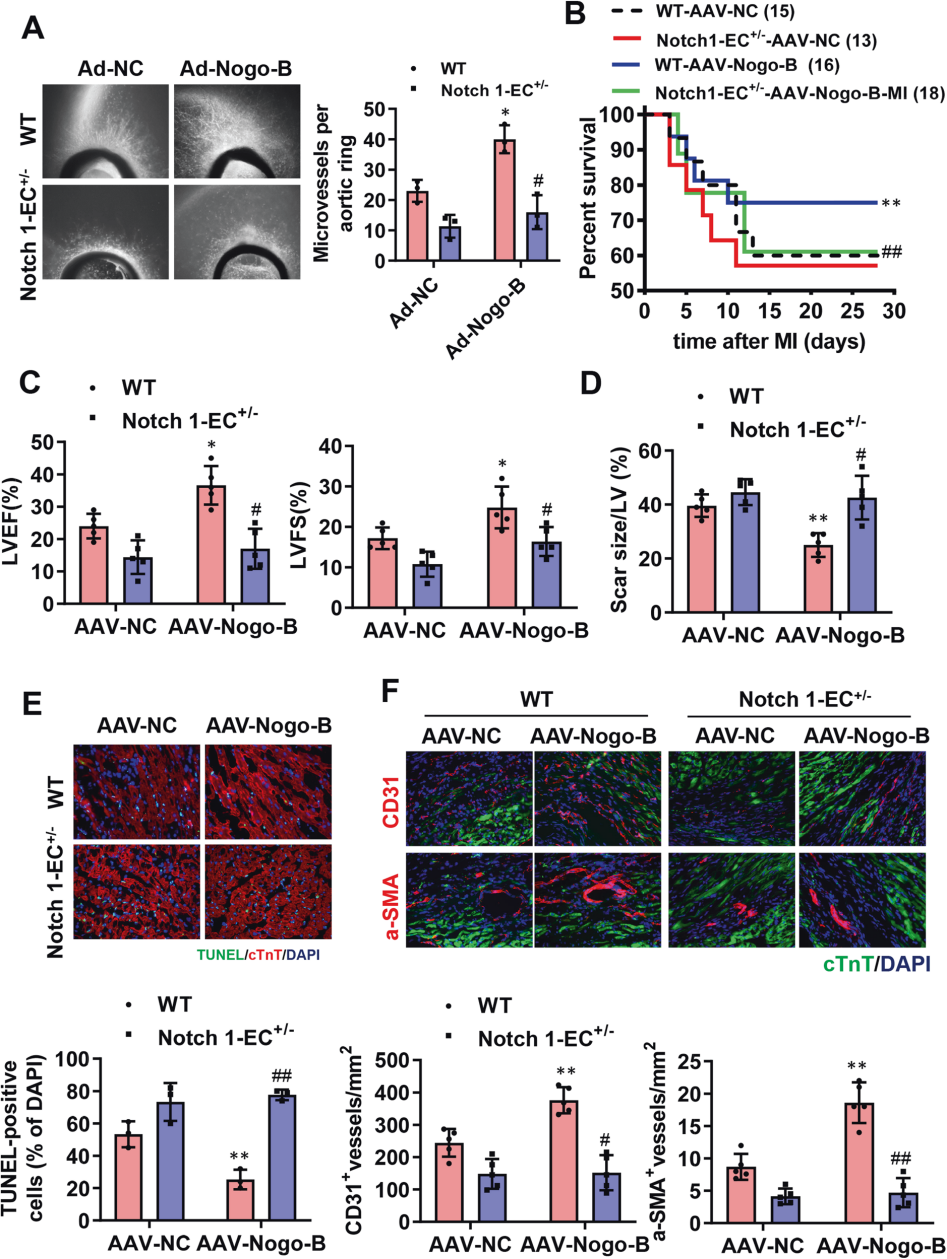
Notch pathway inhibition abolished the cardioprotective effect of Nogo-B in MI-injured hearts. **A** The vessel outgrowth in aortic rings of Nogo-B overexpressed and endothelial cell-specific Notch1 heterozygous (Notch1-EC^+/−^) mice or littermates (*n* = 3). ^*^P < 0.05 versus Ad-NC group. ^#^*P* < 0.05 versus Ad-Nogo-B group. **B** The survival rate of Notch1-EC^+/−^ and Ctrl mice injected with AAV-Nogo-B and AAV-NC after MI. The log-rank test demonstrated significant differences between the two survival curves. **C** LVEF and LVFS were measured by echocardiography (*n* = 5). **D** Quantitative data of hearts stained with Masson’s trichrome at day 28 post-MI (*n* = 5). **E** Representation and quantification of IHC staining for TUNEL + cardiomyocytes in the border zone of infarcted hearts at day 3 post-MI (*n* = 3). **F** Immunochemistry staining for CD31 + endothelial cells and α-SMA + vessels at day 28 post-MI (*n* = 5). Data were expressed as mean ± SEM. ^*^P < 0.05, ^**^P < 0.01 versus AAV-NC group. ^#^*P* < 0.05, ^##^*P* < 0.01 versus AAV-Nogo-B group^.^ two-way ANOVA (**A** and **C**–**F**).

To further investigate the role of Notch in the cardioprotective effects of Nogo-B, specific endothelial Notch1 heterozygous (Notch1-EC^+/−^) mice were generated. At baseline, the endothelial Notch1 heterozygous mice exhibited normal cardiac phenotypes and functions (Supplementary Fig. 5). Consistent with the above in vitro results from DAPT treatment, Notch1-EC^+/−^ also reduced the cell network/capillary formation of Nogo-B-overexpressed aortic rings (Fig. 8A). Those results demonstrate that inhibiting the Notch pathway can restrain angiogenesis induced by Nogo-B. To further explore the role of the Notch pathway in cardioprotection conferred by Nogo-B in vivo, AAV-Nogo-B and AAV-NC were injected in Notch1-EC^+/−^ and WT mice hearts 4 weeks before MI and the survival rate (Fig. 8B), cardiac function (Fig. 8C), scar size (Fig. 8D), apoptosis of cardiomyocytes (Fig. 8E), and angiogenesis (Fig. 8F) were assessed after MI. Nogo-B induced increases in LVEF, LVFS, survival rate, and angiogenesis and decreased apoptosis and scar size in Notch1-EC^+/−^ mice (Fig. 8B–F). These results imply that the Notch pathway mediates pro-angiogenesis of Nogo-B, therefore, mediating the cardioprotective effect of Nogo-B (Supplementary Fig. 6).

## Discussion

In this study, we reported that the expression level of Nogo-B was upregulated during MI, and Nogo-B overexpression ameliorated heart dysfunction and fibrosis and decreased cardiomyocytic apoptosis after MI, suggesting that Nogo-B is instrumental in cardiac repair. We further demonstrated that Nogo-B promoted angiogenesis in vitro and in vivo. Mechanistically, the Notch signaling pathway mediated the cardioprotective effects of Nogo-B. Through the inactivation of Notch by an inhibitor or specific endothelial heterozygous knockdown, we revealed that the Notch signaling pathway is pivotal for Nogo-B to regulate angiogenesis in MI. Also, some limitations must be considered for the interpretation of our results. It is widely known that the use of anesthesia can affect cardiac function. The influence of anesthetic agents on the infarction process in the ischemic myocardium in our study is unclear.

Nogo-A is expressed in the brain, spinal cord, eye, and skeletal muscle, and most of our knowledge on Nogo-A concerns its function as a neurite outgrowth inhibitor in the central nervous system [24]. Nogo-C is the shortest one among three Nogo isoforms, which expresses in multiple tissues and cells. Nogo-C is upregulated in ischemic myocardium and hypoxic cardiomyocytes and Nogo-C deletion confer cardioprotective effects via alleviating cardiomyocyte apoptosis [25]. Moreover, Nogo-C was upregulated in cardiac fibroblast during MI, which contributes to cardiac fibrosis via interacting and stabilizing ER Ca^2+^ leakage channel Sec61α [26]. However, it is not clear whether Nogo-C functions in cardiac endothelial cells or participates in angiogenesis induction post-MI. Nogo-B is a conserved ER protein and functions to maintain the ER structure [13, 26, 27]. Much evidence described that Nogo-B could regulate cardiovascular diseases, such as cardiac hypertrophy, atherosclerosis, and ischemia [11, 28, 29]. Nogo-B plays a vital role in cardiovascular physiology, and an increase in Nogo-B expression in blood vessels can attenuate vascular inflammation and remodeling [30, 31]. Moreover, angiogenesis is instrumental in cardiac regeneration after myocardial infarction. Nogo-B has been demonstrated to be essential in regulating endothelial cell activities and vascular function in mice [32]. The influence of Nogo-B in patients with cardiovascular disease has been also revealed [33]. However, whether Nogo-B can preserve cardiac functions after MI by promoting angiogenesis remains to be elucidated. Our study further exposed that Nogo-B exerted a role in regulating endothelial cell activities in the MI model, thus supplementing the in vivo data to the angiogenesis regulatory potential of Nogo-B.

Current evidence has highlighted the critical role of Notch signaling in the development of cardiovascular diseases, including MI and hypertrophy [34–36]. Recent studies indicated that Jagged1-mediated Notch1 signaling is involved in cardiac repair during MI [37]. In the adult myocardium, Notch1 signaling is quiescent under normal physiological conditions, while it’s transiently activated following myocardial ischemic injury or other stresses [38]. Moreover, both Notch1 and Jagged1 are expressed in endothelial cells, wherein they play a key role in sprouting angiogenesis and modulate revascularization in ischemia-related disease [39, 40]. The interaction between Notch receptors and their ligands leads to proteolytic cleavage and releases the Notch intracellular domain (NICD). The latter then translocates to the nucleus to activate the transcription of target genes, including Hes1, which has been reported to be highly involved in angiogenesis [41, 42]. In this study, we found that Notch and Hes1 were upregulated after MI and were further increased in Nogo-B overexpressed endothelial cells and hearts. Activation of Notch1 could induce blood vessel network growth, sprouting, branching, and the proliferation of endothelial cells [34–36]. Consistent with previously mentioned studies, our data demonstrated that Nogo-B upregulation increased angiogenesis in vitro and in vivo after MI, and these effects were reversed by the Notch inhibitor. These results infer that Notch mediates the angiogenic regulation of Nogo-B. Taken together, our study demonstrates that Nogo-B increases angiogenesis after MI by activating the Notch signaling pathway.

In conclusion, this study illustrates Nogo-B’s function and mechanism in angiogenesis after MI. Overexpression of Nogo-B after MI may thus promote angiogenesis and cardiac repair. To conclude, Nogo-B may also have therapeutic implications for promoting regeneration in the context of ischemic vascular disease.

## Materials and methods

### Animals

*VE-cadherin-Cre* (*Vecad*-Cre) (C57BL/6 J background) and *Notch1*^flox^ (C57BL/6 J background) mice were obtained from the GemPharmatech Co. Ltd. *Notch1*^flox^ mice were bred with *Vecad*-Cre mice to produce *Vecad*-Cre+; *Notch1*^flox^ heterozygote mice and littermates *Vecad*-Cre-; *Notch1*^flox^ heterozygote mice. All procedures involving animals were performed in accordance with the Guidelines for Care and Use of Laboratory Animals published by the United States National Institutes of Health and were approved by the Institutional Animal Care and Use Committee of Shanghai University of Health and Medicine. Mice were sacrificed with an overdose of anesthesia with 1% pentobarbital sodium (100 mg/kg, i.p.) at the indicated time point.

### CMEC isolation and oxygen-glucose deprivation (OGD) treatment

CMECs were isolated from myocardial tissues using CD31-coupled microbeads as previously described [43] and cultured in an endothelial cell medium (ECM) (ScienCell, #1001), which consisted of basal DMEM, supplemented with 5% fetal bovine serum (FBS, ScienCell, #0025), 1% endothelial growth factor (ECGS, ScienCell, #1052), and 1% penicillin/streptomycin solution (P/S, ScienCell, #0503).

### Adenovirus and adeno-associated viral vector construction, infection, and injection

Adeno-associated viruses expressing human Nogo-B cDNA for Nogo-B (AAV9- Nogo-B) with the mouse endothelial-specific promoter Tie2 were prepared as previously described [44]. About 1 × 10^12^ vg of AAV9-Nogo-B or AAV9-NC were injected intravenously into the tail veins of 4–5 weeks old male C57 mice, as previously described [45]. Sham or surgical treatment of acute myocardial infarction was conducted 4 weeks after AAV9 injection.

Nogo-B cDNAs and short hairpin RNAs of Nogo-B were constructed as described previously [46]. One day after plating, CMECs were incubated for 2 h with recombinant adenoviruses. After removing the viral suspension, cells were replaced in the maintenance medium for 2 days and then for other stimuli, as indicated. Adenoviral transduction at a multiplicity of infection of 20 did not induce significant cell death.

### RNA isolation and quantitative PCR

Total RNA was isolated from the cardiac tissues with Trizol Reagent (Invitrogen, Carlsbad, CA, USA). Relative quantitation by real-time PCR involved SYBR Green detection of PCR products in real-time with the ABI PRISM 7700 Sequence Detection System (Applied Biosystems). GAPDH RNA was amplified as a reference standard. The reactions were performed in triplicate by heating the reactant to 95 °C for 5 min, followed by 40 cycles of 94 °C for 30 s, 58 °C for 30 s, and 72 °C for 30 s, respectively. Primers were as follows: Nogo-B forward: GTTGACCTCCTGTACTGGAGA, Nogo-B reverse: CTGTTACGCTCACAATGCTGA; VEGF forward GCCAGCACATAGGAGAGATGA, VEGF reverse CAAGGCCCACAGGGATTTTCT; GAPDH forward CAAATTCCATGGCACCGTCA, GAPDH reverse GGAGTGGGTGTCGCTGTTGA.

### Western blot analysis

Using RIPA lysis buffer (with protease and phosphatase inhibitor, Thermo Scientific), cells or the hearts were harvested on ice. Protein samples were prepared for PAGE gel electrophoresis, transferred to PVDF membranes (BioRad), and blocked in 4% BSA. The membranes were incubated with primary antibodies overnight at 4 °C, followed by incubation with secondary antibodies at room temperature for 1 h. The following antibodies were used in this study: Nogo-B (R&D, AF6596), GAPDH (Cell signaling technology, CST#5174), Notch1 (Abcam, ab52627), and Hes1 (Abcam, ab119776).

### In vivo model of myocardial infarction

The 10–12 weeks old C57BL/6 male mice were anesthetized via intraperitoneal injection of 50 mg/kg sodium pentobarbital, ventilated (isoflurane 1–2% vol/vol) with a volume-regulated respirator (SAR830, Cwe Incorporated), and MI was induced through permanent ligation of the left anterior descending (LAD) coronary artery with an 8-0 Prolene suture, while mice from the sham group had a loose suture placed in the same position.

### Heart function measurements

Transthoracic echocardiography (Vevo 2100, Visual Sonics) with a 25-MHz imaging transducer was performed on 2.0% isoflurane-anesthetized mice to measure cardiac functions with the M-mode at day 28 after surgery. All measurements were averaged from three consecutive cardiac cycles.

The pressure–volume loops were determined using a pressure–volume loop as previously described [47]. After echocardiography, a polyethylene pressure Millar transducer catheter was inserted from the right carotid artery into left ventricular (LV) for hemodynamic study. Hemodynamic index, including LVDP, LVEDP, LV maximum ascending rates of LV pressure (+dp/dt max), and SV were recorded simultaneously. Chart software (AD Instrument Ltd., Australia) was used for data processing.

### Masson’s trichrome staining

For the Masson’s trichrome stained images, morphometric parameters in each heart, including total LV area and scar area, were blindly analyzed on three slices per heart, and the scar size was calculated as the total scar area divided by the LV area, as measured with Image J.

### Immunohistochemical (IHC) staining

IHC analysis was performed as previously described. In short, extracted hearts from the anesthetized mice were rapidly embedded in OCT (SAKURA) compound after washing with cold cardiac arresting buffer (10% KCl), PBS, and 4% paraformaldehyde. Cardiac tissues were then sliced into five sections (5 µm) each from ligated to the apical position of the heart at 400 µm intervals, and the slices were frozen at −80 °C for subsequent histological analyses. For fluorescent IHC analysis, fresh frozen slices were fixed with 4% paraformaldehyde, permeabilized in 0.4% Triton X-100 (Sigma), and stained with primary antibodies against cardiac troponin T (cTnT, 1:500, ab8295), CD31 (1:200, ab28364), and α-smooth muscle actin (α-SMA, 1:500, ab5694) (Abcam), respectively. Fluorescent conjugated secondary antibodies were used to detect primary antibodies. Nuclei were stained with DAPI. Three microscopic fields of border areas in LV were quantified for each section using Image J.

### Terminal deoxynucleotidyl transferase dUTP nick end labeling (TUNEL) staining

The in situ Cell Death Detection Kit (Roche Applied Science, Germany) was employed to measure TUNEL positive (TUNEL+) cardiomyocytes in MI hearts. The percentage of TUNEL + cardiomyocytes was blindly quantified as the ratio of TUNEL + cardiomyocytes to the total cardiomyocytes, as described previously [5].

### Migration assay

Cell migration assay was performed as previously reported [48, 49]. In brief, 100% confluent monolayers of CMECs were treated with 10 μg/mL mitomycin (Sigma M0503) for 2 h, scratched, and washed three times with PBS. The scratched areas were captured using a Zeiss inverted microscope at 0 and 24 h after different treatments.

### Tube formation assay

For the tube formation assay, Growth factor‐reduced Matrigel (BD Biosciences) was added to 24‐well culture plates (200 µl per well), coated with Corning Matrigel Basement Membrane Matrix Growth Factor Reduced (Corning), and incubated at 37 °C for half an hour. A total of 5 × 10^4^ CMECs in 500 µl of EGM‐Plus medium supplemented with 0.1% fetal bovine serum were added to each well. Capillary-like structure formation was recorded using a Zeiss inverted microscope. Tube length was measured by Image J [50].

### Ex vivo aortic ring assay

As previously described, the thoracic aortas were collected from adult male C57BL/6 mice [51]. Dissected aortas were cut into cross-sectional rings (1–1.5 mm in length) in 24 well plates, embedded in growth factor reduced Matrigel (BD Bioscience), and then incubated at 37 °C for 30 min, overlaid with 0.5 mL of an MCDB131 medium containing 1% FBS and the adenovirus. The rings were incubated at 37 °C for 7–10 days, and images were acquired daily to examine vessel sprouting. The maximum distance from the aortic ring body to the end of the vessel sprout (sprout length) was measured at three different points in each ring.

### RNA-seq

Total RNAs were extracted from CMECs of infarct hearts infected with AAV-NC and AAV-Nogo-B at 1-week post-MI with TRIzol (TIANGEN, DP405). Reverse transcription was performed using a SMARTer Ultra Low RNA Kit (Clontech). cDNA was amplified using an Advantage 2 PCR Kit (Clontech) according to the manufacturer’s protocol. Next, cDNA libraries were constructed using the KAPA Stranded mRNA Seq Kit (KAPA) as the manufacturer’s instructions. Sequencing was performed on Illumina HiSeq2500 with paired-end 150 bp read length.

### Statistical analysis

Data were expressed as mean ± SEM and performed with the Graphpad Prism software (version 8.0). Animals were randomly assigned to experimental groups. For animal studies, no data points were excluded. Sample sizes of all experiments were predetermined according to our experience. Data distribution was assumed to be normal, but this was not formally tested. The investigators were blinded for the mice’s genotype during surgery, echocardiography, organ weight determination, and histological and immunofluorescence quantifications. Statistical significance was compared via paired the Student’s *t*-test or analyzed by one- or two-way analysis of variance (ANOVA) followed with Bonferroni’s multiple as appropriate.

## Supplementary information

Supplementary figure

Reproducibility checklist

## Data Availability

All data needed to evaluate the conclusions in the paper are present in the paper. Additional data related to this paper may be requested from the corresponding author.
