# Supplementary material for: Nogo-B promotes angiogenesis and improves cardiac repair after myocardial infarction via activating Notch1 signaling
Source: Cell Death Dis. 2022 Apr 5;13(4):306. doi: 10.1038/s41419-022-04754-4 (PMC8983727; doi:10.1038/s41419-022-04754-4)
Supplement: Supplementary file 1 — Supplementary figure [file 41419_2022_4754_MOESM1_ESM.pdf]

Nogo-B promotes angiogenesis and improves cardiac repair after myocardial infarction via activating Notch1 signaling

Yanjun Zheng[1, 4], Jingrong Lin[2,4], Dingsheng Liu[1], Guoqing Wan[1], Xuefeng Gu[1], Jian Ma[3, *]

[1] Shanghai Key Laboratory of Molecular Imaging, Zhoupu Hospital, Shanghai University of Medicine and Health Sciences, Shanghai 201318, China

[2]Department of Hypertension, Ruijin Hospital, Shanghai Institute of Hypertension, Shanghai Jiao Tong University School of Medicine, Shanghai, China

[3]Department of Cardiology, Shanghai Jiao Tong University Affiliated Sixth People's Hospital, No. 600. Yi Shan Road, Shanghai, 200233, China.

[4]These authors contributed equally: Yanjun Zheng, Jingrong Lin.

[*]Correspondence to: Jian Ma, Department of Cardiology, Shanghai Jiao Tong University Affiliated Sixth People's Hospital, No. 600. Yi Shan Road, Shanghai, 200233, China.

Email: mjyww2009@126.com

SUPPLEMENTARY METHODS

Identification of the cultured CMECs

Immunofluorescence was used to identify the cultured CMECs. Briefly, cells were fixed with 4% paraformaldehyde and permeabilized with 0.5% Triton X-100. After that, the cells were incubated with pre-diluted primary antibodies against CD31 and vWF overnight at 4°C, followed by 1 hour at room temperature with fluorescent secondary antibodies. The images were visualized with a fluorescence microscope. The flow cytometry was also utilized to identify the purity of cultured CMECs with CD31-PE antibody. The cell precipitates were resuspended by 100 μl PBS and finally detected in a flow cytometer.

Isolation of adult cardiomyocytes, cardiac fibroblasts, and CMECs.

Adult cardiomyocytes were isolated using the Langendorff-perfused method. For isolation of non-myocyte enriched cells, hearts were dissected free of vessels and atria, washed in ice-cold modified Krebs-Henseleit bicarbonate (KHB) buffer (pH 7.2), and quickly cut into pieces. The heart pieces were incubated in 5 ml of digesting solution (0.25 mg/ml Liberase TH [Roche] and 10 mM HEPES in balanced salt solution containing calcium and magnesium) for 8 minutes at 37°C with vigorous stirring. The supernatant was then added to 10 ml of ice-cold KHB. Five milliliters of fresh digesting solution were added to the remaining tissue fragments, and the digestion and sampling steps were repeated until all the tissue was dissolved. The collected cells were filtered through 35-μm nylon mesh (BD Falcon) and then used for flow cytometry. Fibroblasts and ECs were sorted from non-myocyte-enriched cell populations using anti-Thy1 antibody for fibroblasts and anti-CD31 for ECs.

Suppl. Fig. 1

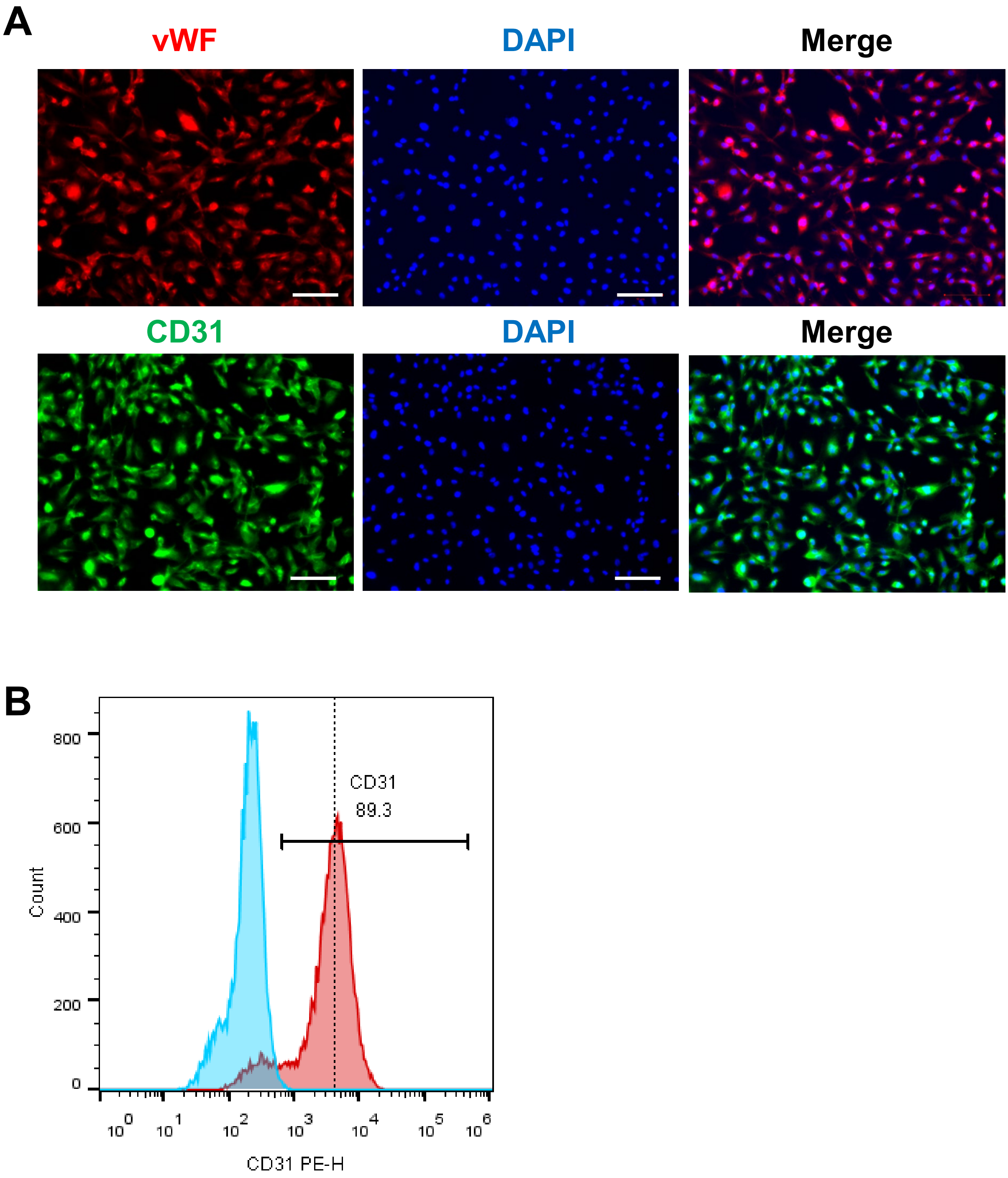

Suppl. Fig. 1. Representative immunofluorescence images showing Von Willebrand Factor (vWF) (upper panel) and CD31 (lower panel) expression in cultured rat cardiac microvascular endothelial cells (CMECs). Images were acquired from five random microscopic fields per group. B, Representative flow cytometry images showing CD31-PE expression of isolated CMECs. Scale bar = 10μm.

Suppl. Fig. 2

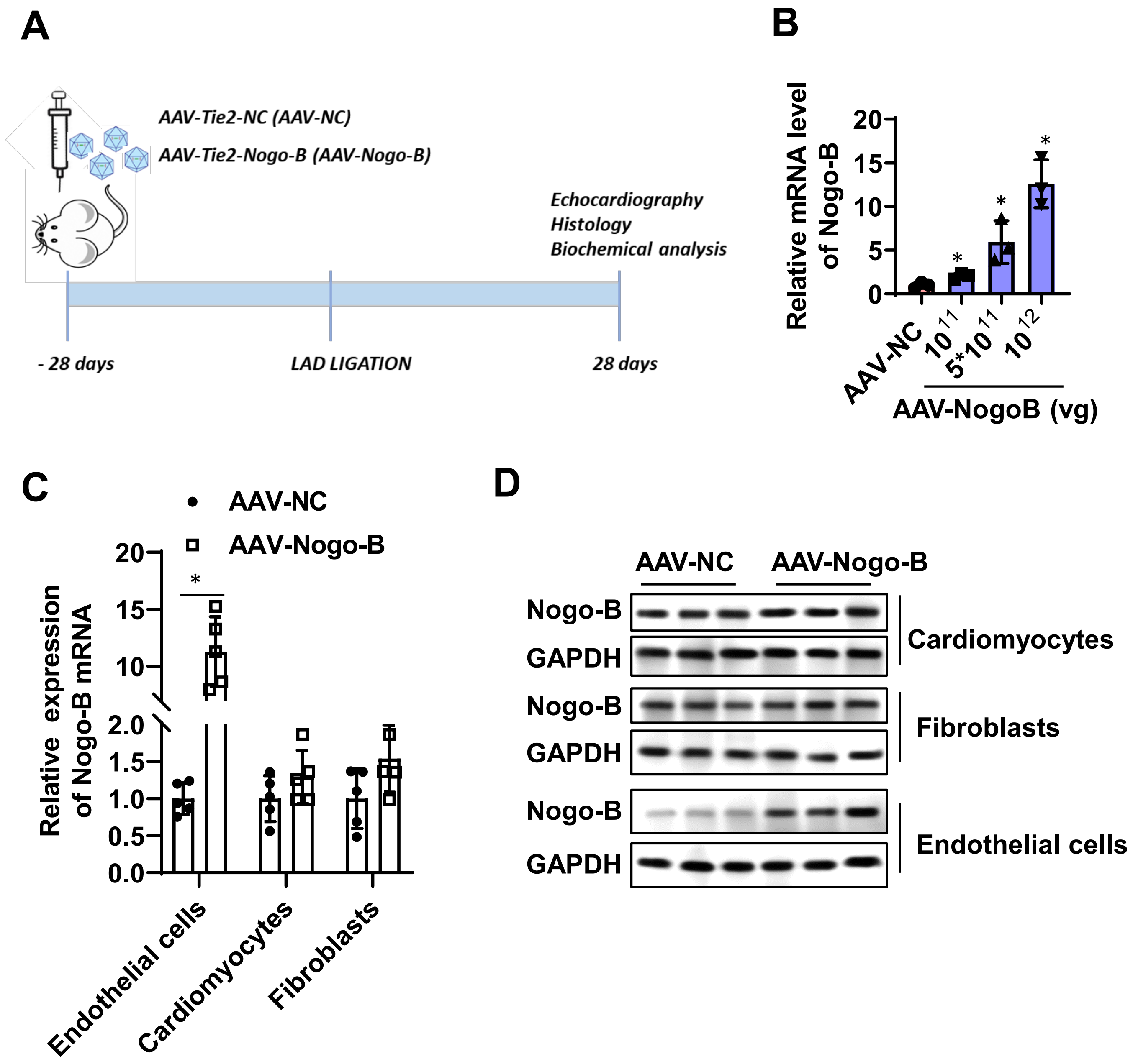

Suppl. Fig. 2. (A) Timeline of AAV9-mediated overexpression experiment: 28 days before induction of MI via a permanent LAD ligation, wild-type mice were injected with either AAV9-Nogo-B or Control-AAV9 (AAV-NC) vector (both under the control of a Tie2 promoter). (B) Dose-dependent Nogo-B overexpression could be detected after 28 days in isolated CMECs (n=3) . Data are the mean± SEM.***P* < 0.01 versus AAV-NC group. one-way ANOVA (B). C-D. mRNA level (A) and protein level (B) of Nogo-B in isolated cardiomyocytes, cardiac fibroblasts, and ECs from adult mice 2 weeks after AAV injection. Data are expressed as mean± SEM. **P* < 0.05.

Suppl. Fig. 3

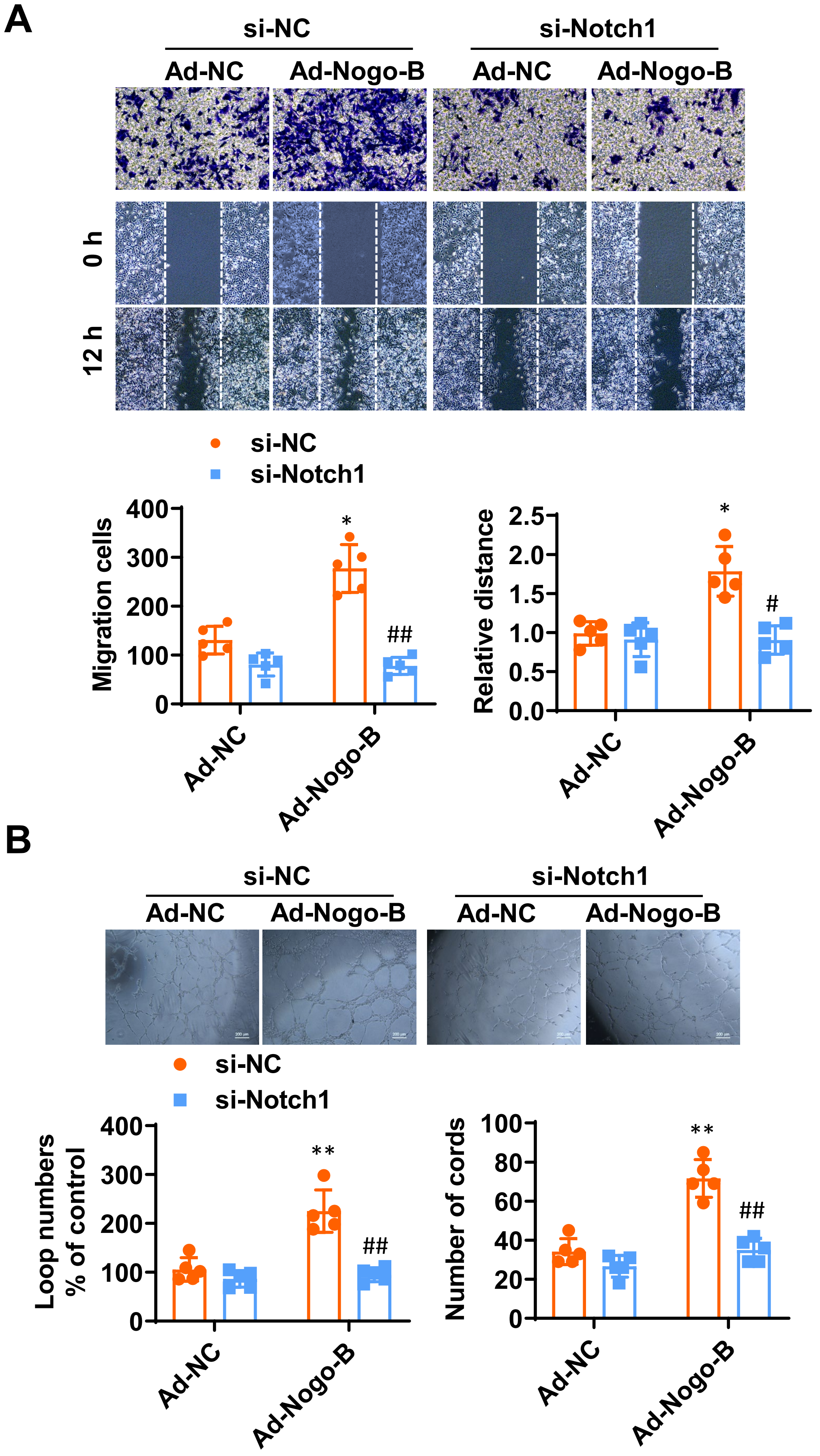

Suppl. Fig. 3. (A) Transwell and scratch/wound assay, and tube formation analysis of Ad-Nogo-B and si-Notch infected Ecs (n=5). (B) Effect of Ad-Nogo-B and si-Notch on vessel out growth in aortic rings and ECs tube formation (n=5). Data are the mean± SEM. $^{*}P < 0.05$, $^{**}P < 0.01$, $^{***}P < 0.001$ versus Ad-NC group. $^{\#}P < 0.05$, $^{\#\#}P < 0.01$ versus Ad-Nogo-B group. two-way ANOVA (A-B).

Suppl. Fig. 4

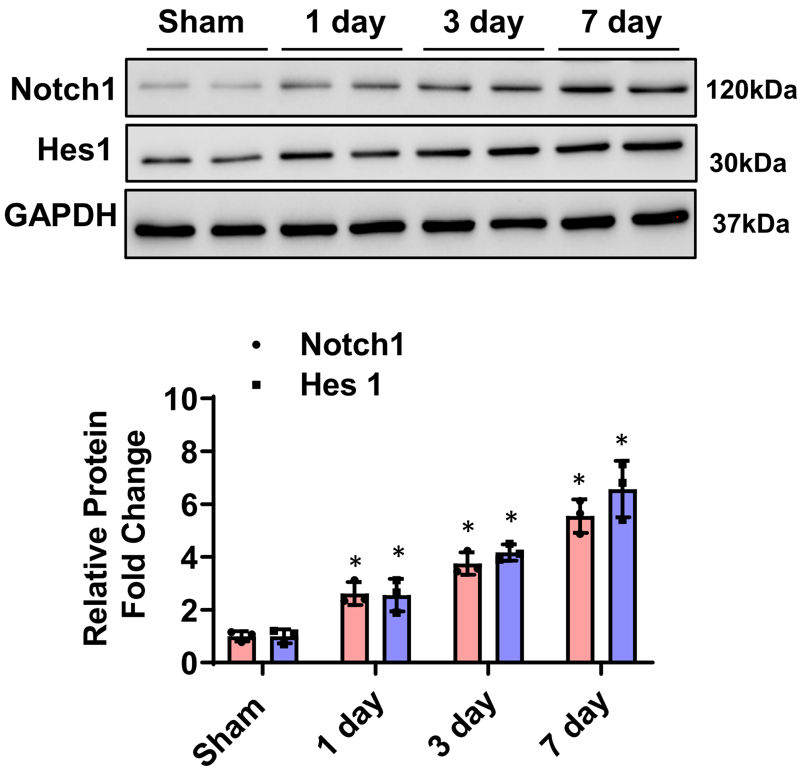

Suppl. Fig. 4. Protein levels of Notch and Hes-1 in Sham and MI hearts. Data are the mean± SEM. **P* < 0.05 versus sham group. one-way ANOVA .

Suppl. Fig. 5

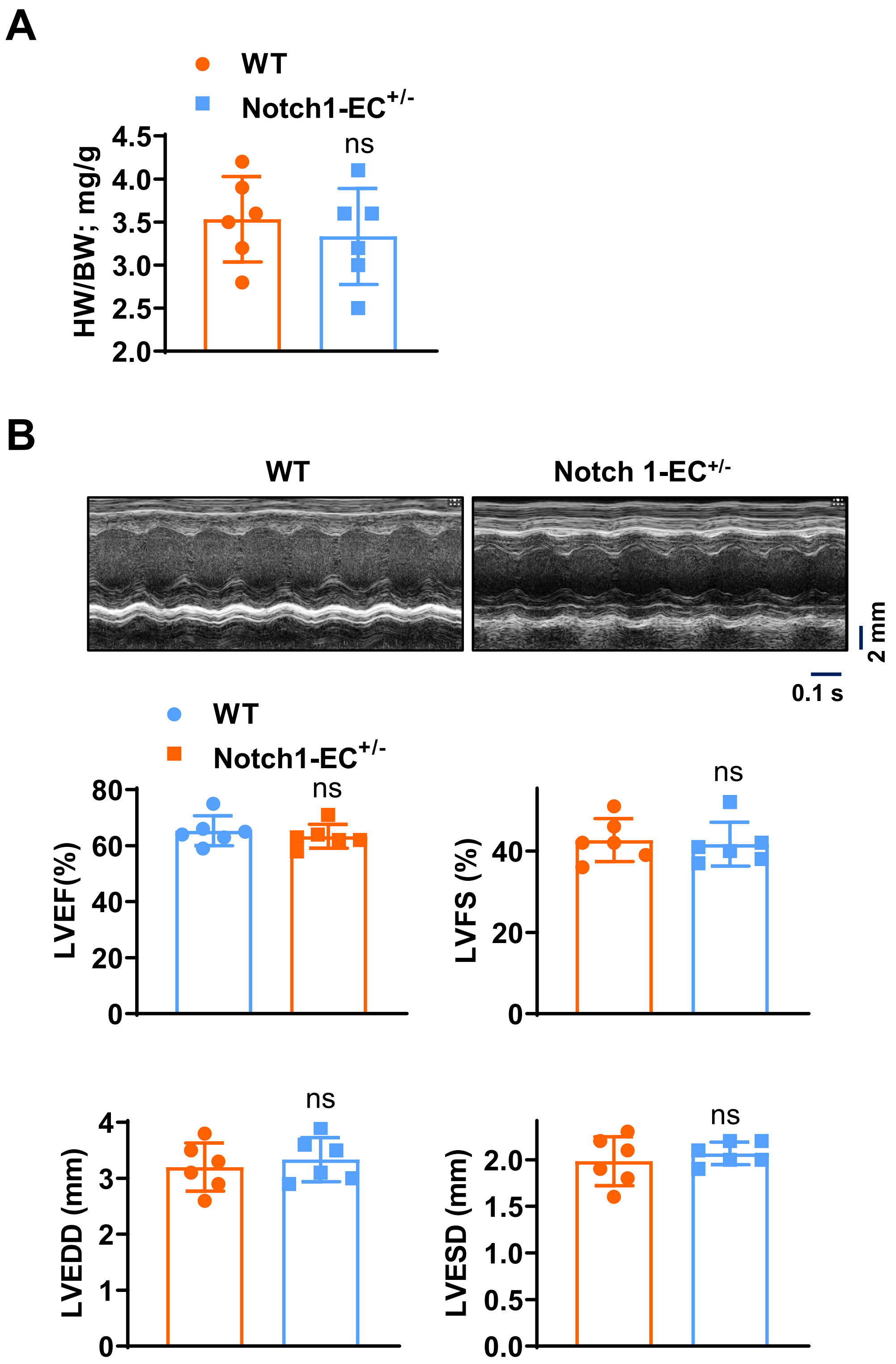

Suppl. Fig. 5. (A) Heart weight/Body weight of specific endothelial Notch1 heterozygous (Notch1-EC$^{+/-}$) mice and littermates Vecad-Cre-; Notch1flox/flox (WT) mice. (B) LVEF, LVFS, LVEDD and LVEDS measured by echocardiography of Notch1-EC$^{+/-}$ and Ctrl mice. ns, no significant. unpaired Student's t-test.

Suppl. Fig. 6

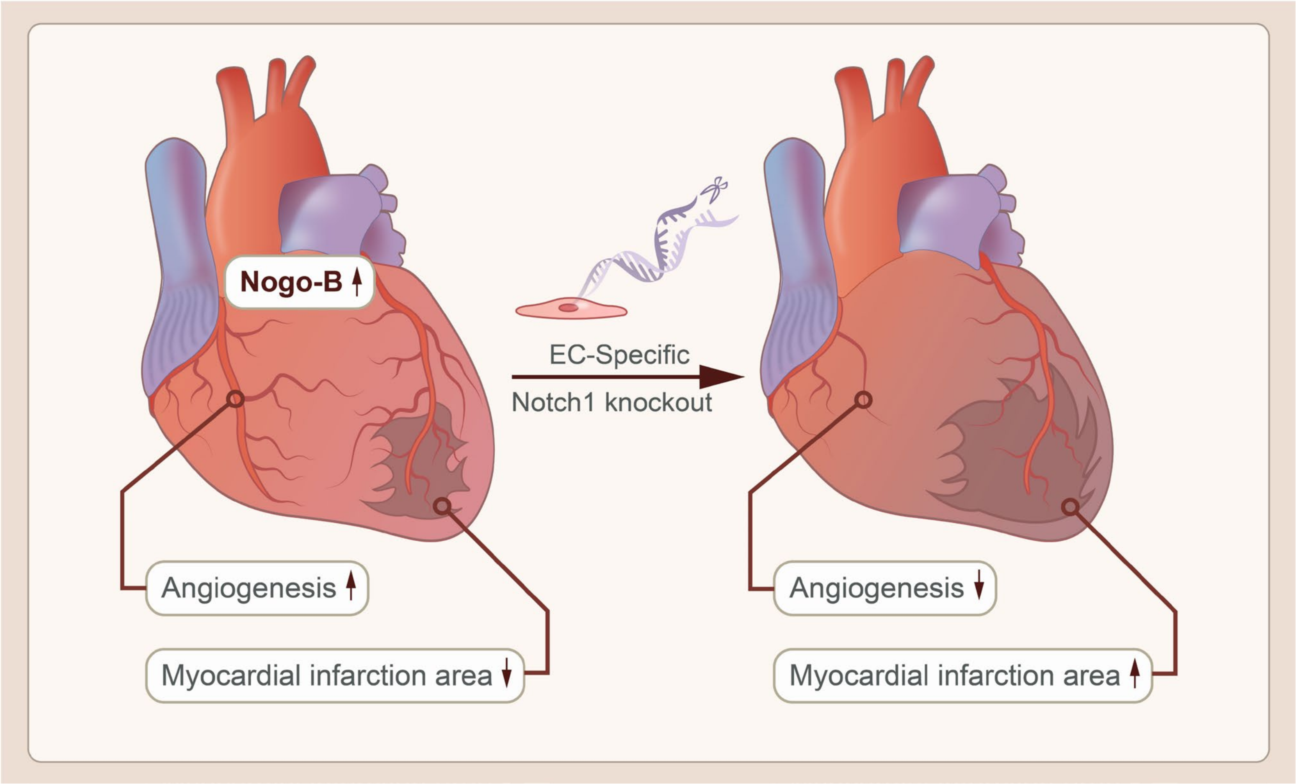

Suppl.Fig.6. Schematic illustration of Nogo-B-Notch1-angiogenesis in myocardial infarction .
